# Editorial: Extremophiles in Lignocellulose Degradation

**DOI:** 10.3389/fmicb.2022.915291

**Published:** 2022-05-04

**Authors:** Aicha Asma Houfani, Mirko Basen, Daniel G. Olson, Sara E. Blumer-Schuette

**Affiliations:** ^1^Michael Smith Laboratories and Department of Biochemistry and Molecular Biology, University of British Columbia, Vancouver, BC, Canada; ^2^Faculty of Mathematics and Natural Sciences, Institute of Biosciences, University of Rostock, Rostock, Germany; ^3^Thayer School of Engineering, Dartmouth College, Hanover, NH, United States; ^4^Department of Biological Sciences, Oakland University Rochester, Rochester, NY, United States; ^5^Department of Bioengineering, Oakland University Rochester, Rochester, NY, United States

**Keywords:** extremophiles, lignocellulose, extremozymes, enzyme-oriented discovery, CAZymes

Cellulose, the major compound of the cell walls of plants and algae, is the most abundant polymer on earth. Algal biomass as well as lignocellulose from land plants hence represent an inexpensive renewable energy source. Extreme environments are attractive ecosystems for the isolation of heat- or salt-tolerant enzymes from (hyper)thermophilic or halophilic microorganisms, respectively (Schröder et al., 2020). These enzymes are relevant to the bioenergy sector as well as to other industries in the production of paper food and other crops, and textiles. Since the biodiversity in extreme habitats is far from well-known, researchers continue to prospect for novel and/or industrially relevant “extremozymes,” and extremophiles themselves as biocatalysts (Elleuche et al., 2014; Sarmiento et al., 2015). Both of which have been featured in this Research Topic, about the decomposition and conversion of lignocellulosic biomass.

Both culture dependent and metagenomic approaches continue to be relevant methods for the discovery of “extremozymes.” With the reduction in sequencing costs, metagenomics is becoming increasingly important in enzyme-oriented discovery projects on sustainability themes, contributing to the identification of widely distributed, extremophilic microorganisms encoding Carbohydrate-Active enZymes (CAZymes) (Strazzulli et al., 2020). This was demonstrated in the study by Reichart et al. where the authors analyzed publicly available metagenomes representing 58 individual hot springs or geothermal features. Using bioinformatics tools and computational strategies, the authors detected a significant correlation between microbial diversity and temperature. Differences between datasets with respect to metadata collection and sequencing methods resulted in no clear correlation between geochemical parameters of a given hot spring and lignocellulose degradation potential. However, the increasingly diverse profile including novel taxa encoding for CAZymes across microbial phylogeny and thermal gradients presents tremendous opportunities for future bioprospecting.

An important area of study looks at mechanisms for adaptation and acquisition of new traits among thermophilic lignocellulolytic microorganisms. A lignocellulose-degrading thermophilic microbial culture was investigated by López et al. at all stages of plant waste composting, paying particular attention to the dynamics, enzymes, and thermotolerance of each community component. In addition to the enzymes that these extremophiles express and their growth temperature range, such details provide significant insights into the evolution of thermophilic microbes during the composting process. Thermostability studies are also encouraged when enzymes are produced by thermophilic microorganisms. Chen et al. explored hydrothermal deep-sea sediments to capture and characterize a thermostable β-Glucosidase from the archaeon *Thermofilum* sp. (TsBGL), exhibiting the highest activity at 90°C for 1.5 h, and thus being comparatively more active and more thermally tolerant than mesophilic β-glucosidases. By providing TsBGL as an enzyme of choice for the development of enzymatic cocktails utilized in biological applications, the enzyme opens the door to a range of biotechnological and industrial applications.

Fungi are widely known to produce the majority of cellulases and xylanases. In many industries, *Aspergillus* endoxylanases are used due to their high enzyme activity production. However, bacterial xylanases have superior properties when it comes to extremophilic properties. Verma offered an overview of bacterial endoxylanases, while also providing insights into the molecular basis of the extremophilic xylanases. In his literature review, the author showed that some properties occur exclusively in bacterial xylanases, while fungal endoxylanases lack those features. The use of both types of xylanases in industry depends on their availability and properties. Other than bacteria and fungi, xylan breakdown is also occurring in extremophiles archaea. Using activity-based protein profiling (ABPP) to identify CAZymes, Klaus et al. described the process of xylan metabolism by functionally characterizing two enzymes involved in a xylanolytic Euryarchaeon *Thermococcus* sp. Strain 2319x1E. Using the ABPP approach with α- or β-glycosidase selective chemical probes, protein identification is not limited by sequence similarity, and it can identify proteins involved in xylan degradation which have no known homology with previously identified proteins. This resulted in the discovery of a novel bifunctional carbohydrate deacetylase (EGDIFPOO_00674) belonging to the GH57 family and a β-glycosidase (EGIDFPOO_00532) that exhibits xylosidase activity.

In extremophilic conditions, resulting from anthropogenic pollution, lignocellulose degradation potential has also been reported. In this article Research Topic, Ran et al. isolated multiple manganese-oxidizing bacteria from soil originating from a former manganese mining operation. From this extreme environment, manganese-oxidizing *Bacillus safensis* strain ST7 was found to grow in high concentrations of heavy metals, and was found to have lignocellulose degradation potential when studying the bacterial strain's transcriptional response to Mn(II) exposure. Based on the expression of genes from strain ST7's transcriptome, the authors identified 26 genes as being related to lignocellulosic degradation.

Metabolic engineering to improve the activity and stability of biocatalysts is often the limiting step before they can be applied to biotechnological applications. Nevertheless, microorganisms that degrade lignocellulose in extreme environments exhibit robust physiological properties which are well-suited for industrial applications. To improve biocatalysts for consolidated bioprocessing (CBP), where the same microbe degrades and ferments lignocellulose, strain improvements may include improvements in lignocellulose degradation, and ethanol production. In this case, it might be necessary to find equally thermostable microorganisms to serve as the genetic reservoir for metabolic engineering of the biocatalyst. As an example, *Thermoanaerobacterium saccharolyticum*, a thermophilic ethanol producer, often serves as the genetic reservoir to metabolically engineer *Clostridium thermocellum*, the cellulose consumer. In a study using a pyruvate kinase (PYK) of *Thermoanaerobacterium saccharolyticum* (TsPYK), an isozyme having an extra C-domain, Fenton et al. identified TsPYK allosteric properties which might influence the biological outcomes observed in metabolic engineering for ethanol production. In many organisms, PYK activity is activated by AMP and ribose-5-phosphate, however this report identifies that some sugar phosphates (IMP and GMP) are allosteric inhibitors. To produce ethanol, TsPYK was used to improve the conversion of PEP to pyruvate in *Clostridium thermocellum*.

In addition, the ability to improve strains of *Clostridium thermocellum* for CBP will require a genome wide view of transcriptional regulation. Hebdon et al. used DNA affinity purification to identify transcription factor (TF) binding sites in this model cellulolytic bacterium. Starting with 90 putative TFs, they found high quality binding motifs for 11 of them, and confirmed sequence-specific binding for 6. Understanding the networks involved in metabolic processes, including regulation of electron and carbon flux, is crucial for consolidated bioprocessing of cellulosic substrates for ethanol and other valuable compounds.

Although the editorial presented a diverse view on the features of these enzymes and the opportunities they will bring, more research is needed among industrial companies to facilitate the path from enzyme discovery to commercialization for efficient use at the biotechnological level. Therefore, further studies are needed to translate those findings into applicable technologies, and this will also ease the screening the researchers are doing up front of how we could make the most out of the existing extremozymes as well as bring to the world unknown and unrevealed extremozymes.

## Author Contributions

AH wrote the first draft. All authors edited and reviewed the manuscript. All authors approved the content of the manuscript.

## Conflict of Interest

The authors declare that the research was conducted in the absence of any commercial or financial relationships that could be construed as a potential conflict of interest.

## Publisher's Note

All claims expressed in this article are solely those of the authors and do not necessarily represent those of their affiliated organizations, or those of the publisher, the editors and the reviewers. Any product that may be evaluated in this article, or claim that may be made by its manufacturer, is not guaranteed or endorsed by the publisher.
